# Transfusion-Transmitted Hepatitis A Virus, France, 2018

**DOI:** 10.3201/eid2801.210403

**Published:** 2022-01

**Authors:** Caroline Lefeuvre, Caroline Lefort, Françoise Boyer, Sophie Le Cam, Lina Mouna, Anne-Marie Roque-Afonso, Hélène Le Guillou-Guillemette, Rafaël Mahieu

**Affiliations:** Angers University Hospital, Angers, France (C. Lefeuvre, F. Boyer, H. Le Guillou-Guillemette, R. Mahieu);; University of Angers, Angers (C. Lefeuvre, H. Le Guillou-Guillemette, R. Mahieu);; Etablissement Français du Sang Centre-Pays de la Loire, Angers (C. Lefort, S. Le Cam);; University of Paris–Saclay, Villejuif, France (L. Mouna, A.-M. Roque-Afonso);; French National Reference Center for Hepatitis A Virus, Villejuif (L. Mouna, A.-M. Roque-Afonso)

**Keywords:** hepatitis A, viruses, blood donor, blood transfusion, hemovigilance, screening, pathogen inactivation, France, hepatitis A virus, *Suggested citation for this article*: Lefeuvre C, Lefort C, Boyer F, Le Cam S, Mouna L, Roque-Afonso A-M, et al. Transfusion-transmitted hepatitis A virus, France, 2018. Emerg Infect Dis. 2022 Jan [*date cited*]. https://doi.org/10.3201/eid2801.210403

## Abstract

We report a transfusion-transmitted hepatitis A virus infection in an immunocompromised patient in France, detected shortly after a transfusion of pathogen-reduced pooled platelets. This case raises questions about the efficacy of donor screening methods. Additional safety measures, such as routine donation screening, should be considered.

Hepatitis A virus (HAV), a common cause of acute viral hepatitis, is a nonenveloped single-stranded RNA virus. Fecal–oral transmission is the most common route of infection (*1*). Despite availability of a vaccine, the recent increase in HAV infection incidence in high-income countries may indicate either new risks associated with migration flows or ongoing outbreaks in the population of men who have sex with men (MSM) (*1*). Because the prevalence of HAV infection is very low in Europe, few countries have included HAV immunization in their general vaccination recommendations (*2*).

The safety of blood transfusions is tightly linked to a rigorous surveillance of donated blood and recognition of emergent or reemergent infectious diseases. However, not all transfusion-transmitted agents are routinely screened by laboratory testing. HAV is considered clinically insignificant in its transfusion-transmissible infection risk. No country requires HAV blood donation screening; however, some countries require donation screening for the manufacturing of plasma-derived products or pooling of plasma for transfusion (*3*,*4*). The donor selection process using donor history questions has been deemed acceptable to prevent HAV from entering the blood supply despite the risk for a prolonged silent viremia phase. Recognized transfusion-transmitted HAV infection is uncommon (*3*); just 1 case of transmission through organ transplantation has been documented to date (*5*).

## The Study

**Figure 1:**
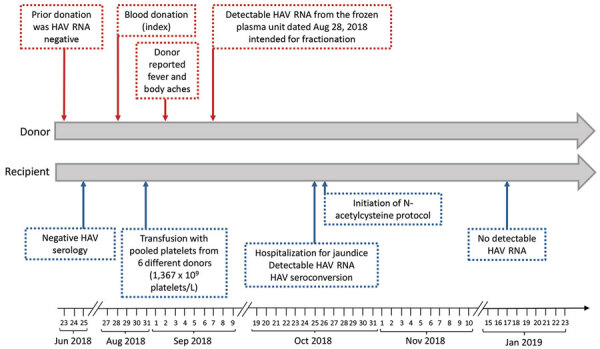
Timeline of events in blood donor and recipient in case of transfusion-transmitted HAV, France, 2018. The donor interview did not reveal any relevant HAV risk factors, including travel, food consumption, personal entourage cases, or unsafe sex practices. The donor was not vaccinated against HAV at the time of donation. The recipient was not vaccinated against HAV at the time of the transfusion; moreover, the recipient has not been vaccinated in postexposure after notification of fresh frozen plasma HAV positivity. The recipient interview reported no other risk factors for HAV, including travel, food consumption, personal entourage cases, and unsafe sex practices, with the exception of recent transfusion. HAV, hepatitis A virus.

We report a transfusion-transmitted HAV infection that was identified by postdonation information in addition to HAV RNA detection from additional quality control of plasma donations intended for use in manufacturing plasma-derived products. The index donation was collected from a male donor on August 28, 2018; after the donor interview and suitable routine screening, it was accepted for use. The donor interview did not reveal any relevant HAV risk factors (Figure 1). Red blood cells, fresh frozen plasma, and pooled platelets (pathogen-inactivated by amotosalen and UV-A light) were manufactured from the donation. On September 2, 2018, the donor reported fever while his donated red blood cells and frozen plasma were still in inventory. The pooled platelets had been transfused to an immunocompromised male patient on August 31, 2018 (Figure 1). Packed red blood cells were immediately destroyed in accordance with the blood bank’s recommendation. The hemovigilance service promptly provided postdonation information to the prescribing physician. HAV RNA was identified from the frozen plasma intended for fractionation on September 7, 2018. A frozen sample from a previous donation from this donor on June 23, 2018, was HAV RNA negative, as were samples from other platelet pool donors.

On October 25, 2018, the recipient of pooled platelets was hospitalized for jaundice. The recipient interview reported no risk factors for HAV infection other than the transfusion on August 31. The recipient’s serum levels of alanine aminotransferase were elevated at 3,553 IU/L. The recipient was reactive for HAV IgM and IgG; he had previously been negative on June 25, 2018. Avidity indexes were <50%, indicating acute HAV infection (Table; Appendix).

**Table:** Laboratory results from donor plasma and recipient serum in case of transfusion-transmitted HAV, France, 2018*

Source	Sample date	HAV test result (s/c)		HAV avidity index, %†	HAV RNA, log_10_ IU/mL	ALT, IU/L
		IgM	IgG			
Donor	2018 Jun 23	NR‡	NR§	ND	<1.00	ND
Donor¶	2018 Aug 28	NR‡	NR§	ND	5.43#	ND
Recipient	2018 Jun 25	NR**	NR††	ND	ND	49
Recipient¶	2018 Oct 25	R** (2.35)	R†† (3.73)	12.95	5.82	3,553
Recipient	2018 Oct 30	ND	ND	ND	ND	1,200
Recipient	2018 Nov 9	ND	ND	ND	ND	144
Recipient	2018 Nov 19	ND	ND	31.85	3.42	ND
Recipient	2018 Dec 10	ND	ND	39.15	3.14	59
Recipient	2019 Jan 17	ND	ND	44.00	<1.00	39

*ALT, alanine aminotransferase; HAV, hepatitis A virus; ND, test not done; NR, nonreactive; R, reactive; s/c, sample/cutoff ratio. †HAV avidity index was expressed as a percentage as follows: HAV avidity index = (the absorbance reading with urea wash/the absorbance reading without urea wash) × 100. Avidity index <50% indicates acute infection. ‡VIDAS Anti-HAV IgM (bioMérieux, https://www.biomerieux.com). Value >0.5 is considered reactive. §Elecsys Anti-HAV total antibodies (value >22 mIU/mL considered reactive) ¶Genotype IA was identified. #Result (log_10_ IU/mL) corresponds to HAV RNA quantification performed at the National Reference Center for HAV. The result of qualitative detection performed at the French National Blood Service was 3.27 (no unit). **ARCHITECT HAV Ab-IgM (Abbott Diagnostics, https://www.diagnostics.abbott). Value >1.20 is considered reactive. ††ARCHITECT HAV Ab-IgG (Abbott Diagnostics). Value >1.00 is considered reactive.

We initiated an N-acetylcysteine protocol on October 26 over 4 days, providing gradual improvement of hepatic symptoms. The HAV viral load was 5.82 log_10_ IU/mL. RNA was still positive 2 months after clinical symptom onset but was no longer detectable on January 17, 2019 (Table), and the recipient made a complete recovery.

**Figure 2:**
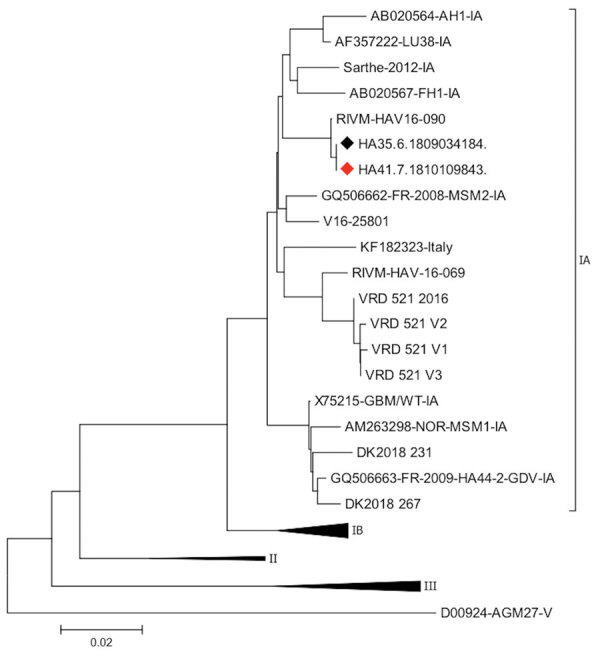
Phylogenetic relationships of viral protein 1/2A sequences in case of transfusion-transmitted hepatitis A virus, France, 2018. Black diamond indicates the sequence from the blood donor; red diamond indicates sequence from the blood recipient. Scale bar indicates nucleotide variation.

We conducted phylogenetic analysis on donor and recipient samples (Appendix). Two sequences from the donor and recipient clustered with the genotype IA and strain RIVM HAV16-090 (Figure 2). The phylogenetic relationships between the sequences provided from the donor and recipient were evidence for HAV transmission by blood transfusion. We identified this case of transmission early, before it was clinically suspected in the immunocompromised recipient. HAV is rarely recognized as a transfusion-transmitted risk, yet has been previously reported (*3*). The transfusion-associated hepatitis A cases previously described were suspected on the basis of a symptomatic recipient or symptomatic contacts.

## Conclusions

HAV identification in blood donation and the recipient, absence of other risk factors in the recipient, and timing of events, in addition to identification of the same strains between the donor and recipient, all confirmed the HAV transmission by blood transfusion. The RIVM HAV16-090 strain was 1 of the 3 strains identified in the 2017 Europe outbreak, which mostly affected MSM in the initial phase before the general population (*6*). During this outbreak, HAV-RNA prevalence was 5-fold higher than that for the 2015–2016 period; the male-to-female sex ratio increased substantially between these 2 periods, from 0.7 to 5.5. 

This outbreak highlights the risks for HAV transmission by transfusion from blood donations collected from MSM (*6*) and also from the general population, even in low-incidence countries. Currently, the risk of collecting blood from an asymptomatic HAV RNA–positive donor is still low in Europe, although sporadic outbreaks occur.

Because of the sporadic nature of acute HAV infections in donors, short viremia phase, and absence of chronic carriers (*7*), HAV RNA screening is not yet recommended at the time of our report (*3*). Nevertheless, for the plasma industry in France, screening by means of HAV nucleic acid testing (NAT) has become a mandatory requirement recommended by the European Pharmacopoeia for plasma donation (*4*), after a series of reported cases (*8*).

Considering the risks of serious infections in immunocompromised patients (*5*), routine additional screening tests for all blood donations by NAT would be useful for both HAV and hepatitis E virus (HEV). Nationwide screening of blood donors for HEV has been introduced in several countries in Europe, including Ireland, the United Kingdom, and the Netherlands (*9*). In France, where HEV is endemic in some areas and some of the population are chronic carriers, a fraction of plasma products to be transfused to high-risk patients has been tested for HEV RNA since late 2012 (*10*).

Other proactive approaches, such as pathogen inactivation (PI) technologies, using physical or chemical methods may prevent the risk for infection transmission. As of April 2019, only inactivation procedures of plasma and platelets have been approved for use in the European Union (*11*). In Europe, 2 systems are routinely used: UV light in combination with a photo-reagent, or a photosensitizer. These systems have been proven ineffective against several nonenveloped viruses, including HAV, parvovirus B19 (*12*,*13*), and HEV. Nonenveloped viruses are able to persist in a blood batch during processing and storage, thereby representing a particular threat to blood safety (*7*). In 2018, a novel PI system for platelet units based on shortwave UV-C light treatment underwent clinical efficacy and safety testing; early testing results suggested that this PI system effectively inactivates HAV in platelet concentrates (*14*). PI cannot be considered a full substitute for blood donor screening for transfusion-transmissible infections like HAV until PI systems for the routine treatment of red blood cells are available. We suggest screening with NAT for HAV, HEV, and parvovirus B19, for which effective PI technologies are not yet available (*13*). The turnaround times for NAT for HAV, HEV, and B19V are comparable to those for other agents for which NAT is applied in screening. Last, timely recognition of postdonation symptoms and notification to blood banks are still paramount to prevent transmission of infectious pathogens not included in routine blood donation screening.

Appendix Additional information about a transfusion-transmitted hepatitis A infection that was not prevented by pathogen inactivation system.
