# Supplementary material for: Transfusion-Transmitted Hepatitis A Virus, France, 2018
Source: Emerg Infect Dis. 2022 Jan;28(1):219–23. doi: 10.3201/eid2801.210403 (PMC8714218; doi:10.3201/eid2801.210403)
Supplement: Appendix — Additional information about a transfusion-transmitted hepatitis A infection that was not prevented by pathogen inactivation system. [file 21-0403-Techapp-s1.pdf]

Article DOI: https://doi.org/10.3201/eid2801.210403

Transfusion-Transmitted Hepatitis A Virus, France, 2018

Appendix

Methods

Laboratory Specimen Collection and Testing

Biochemical and Serological Tests

Testing for alanine aminotransferase (ALT) is not recommended for blood donor screening in France. The recipient patient's ALT was monitored for 84 days after the onset of symptoms (after October 25, 2018).

Serological assays for the donor were performed on serum samples from specimens taken on June 23 and August 28, 2018, with VIDAS Anti-HAV IgM (bioMérieux, https://www.biomerieux.com) and with Elecsys Anti-HAV total antibodies (Roche Diagnostics GmbH, https://diagnostics.roche.com) at the French National Reference Center for HAV, Paul Brousse, Villejuif, France. Serological assays were performed on recipient serum samples with ARCHITECT HAV Ab-IgM and ARCHITECT HAV Ab-IgG on the Architect system Module i2000 (Abbott Diagnostics, https://www.diagnostics.abbott) at the laboratory of virology in Angers University Hospital, Angers, France.

Anti-HAV IgG avidity was measured with an in-house assay as described elsewhere (*1*) at the National Reference Center for HAV (specimens from October 25, November 19, and December 10, 2018, and from January 17, 2019). Avidity indexes <50% indicate acute infection.

HAV Molecular Analysis

First qualitative detection of the HAV-RNA in fresh frozen plasma intended for fractionation was performed using Procleix HAV Assay (Grifols Diagnostic Solutions Inc., https://www.diagnostic.grifols.com) on the Procleix Tigris system at the French National Blood Service (Etablissement Français du Sang Centre – Pays de la Loire [EFS CPDL]). Thus, the

Laboratoire Français du Fractionnement et des Biotechnologies, the public laboratory in charge of the production of blood-derived products and fractionated plasma products, delegates HAV screening to the French National Blood Service; screening is performed in batches within 4 weeks after the donation according to LFB contractual requirements.

Quantification and sequencing were performed at the National Reference Center for HAV, Villejuif. HAV-RNA was extracted from a 200 µL serum or plasma sample using the easyMAG automated extraction system (NucliSENS easyMAG, bioMérieux). Quantification of HAV-RNA was performed using the RealStar HAV RT-PCR kit (Altona Diagnostic Kit GmbH, https://www.altona-diagnostics.com) with a lower quantification limit of 10 IU/mL. For RNA sequencing, the viral protein 1/amino terminus of 2A (VP1/2A) junction region (508 bp) of the HAV genome was amplified using a one-step RT-PCR (OneStep RT-PCR kit, QIAGEN, https://www.qiagen.com) as previously described (*2*). PCR products were sequenced using the ABI PRISM Big Dye Terminator v3.0 cycle sequencing kit on the automated ABI 3130 (Applied Biosystems, https://www.thermofisher.com).

Phylogenetic analysis of nucleotide sequences was conducted using the neighbor-joining method in MEGA7, including donor and recipient sequences and reference sequences as previously described (*2*). The evolutionary distances were computed using the maximum composite likelihood method. The donor and recipient sequences were logged in the sequence database under the following accession numbers: MW703463 for the donor sequence and MW703464 for the recipient sequence.

References

1. Roque-Afonso A-M, Grangeot-Keros L, Roquebert B, Desbois D, Poveda J-D, Mackiewicz V, et al. Diagnostic relevance of immunoglobulin G avidity for hepatitis A virus. J Clin Microbiol. 2004;42:5121–4. PubMed https://doi.org/10.1128/JCM.42.11.5121-5124.2004

2. Couturier E, Roque-Afonso AM, Letort MJ, Dussaix E, Vaillant V, de Valk H. Cluster of cases of hepatitis A with a travel history to Egypt, September–November 2008, France. Euro Surveill. 2009;14:19094. PubMed https://doi.org/10.2807/ese.14.03.19094-en
